# Frequent Genetic Alterations and Their Clinical Significance in Patients With Thymic Epithelial Tumors

**DOI:** 10.3389/fonc.2021.667148

**Published:** 2021-07-08

**Authors:** Song Xu, Xiongfei Li, Hongyi Zhang, Lingling Zu, Lingqi Yang, Tao Shi, Shuai Zhu, Xi Lei, Zuoqing Song, Jun Chen

**Affiliations:** ^1^ Department of Lung Cancer Surgery, Tianjin Medical University General Hospital, Tianjin, China; ^2^ Tianjin Key Laboratory of Lung Cancer Metastasis and Tumor Microenvironment, Lung Cancer Institute, Tianjin Medical University General Hospital, Tianjin, China; ^3^ Department of Thoracic Surgery, Gansu Provincial Hospital, Lanzhou, China; ^4^ Precision Medicine Center, Tianjin Medical University General Hospital, Tianjin, China

**Keywords:** gene mutations, thymic epithelial tumors, NGS, TP53, TCGA

## Abstract

**Purpose:**

Thymic epithelial tumors (TETs) are relatively rare neoplasms, including thymomas (types A, AB, B1, B2, and B3) and thymic carcinomas (TCs). The current knowledge about the biological properties of TETs is limited due to their low incidence. This study aimed to detect genetic alterations in TETs using next-generation sequencing(NGS) and explore their clinical significance in survival.

**Methods:**

Tumor tissues and clinical data were collected from 34 patients with resected TETs in the Tianjin Medical University General Hospital between January 2011 and January 2019, and 56 cancer-associated genes were analyzed. The data of 123 TETs were retrieved from TCGA, and the information on their clinical and somatic mutations was explored.

**Results:**

The cohort comprised 34 TETs including 17 thymomas and 17 TCs. The NGS results indicated that 73.08% of TCs+type B3 TETs and 37.50% of non-TCs+type B3 TETs each exhibited gene mutations. For patients with type B3/C, TP53 was the most frequent mutation (19.23%), followed by CDKN2A (11.54%). Similarly, in 123 TETs from the TCGA cohort, TP53 mutations were more frequent in patients with type B3/C than in patients with non-type B3/C (11.53% *vs* 3.09%). Further, patients with TET with TP53 mutations in the present cohort and the TCGA cohort had a worse prognosis compared with those without TP53 mutations.

**Conclusions:**

Gene mutation profiles between TCs+type B3 TETs and non-TCs+type B3 TETs were significantly different. The presence of TP53 mutations was more frequent in TCs+type B3 TETs than in non-TCs+type B3 TETs, which was associated with a worse prognosis.

## Introduction

Thymic epithelial tumors (TETs) are relatively rare neoplasms originating from the epithelial cells of the thymus, but they are the most common type among tumors of the anterior mediastinum (1, 2). TETs include a heterogeneous group of rare tumors. The World Health Organization (WHO) and the Masaoka–Koga stage classification are used for the histological classification and clinical staging of these tumors (3, 4). According to the WHO 2015 criteria, TETs are classified into thymomas (types A, AB, B1, B2, and B3) and thymic carcinomas (TCs) depending on the morphology of epithelial cells and the relative amount of thymocytes (3, 4). The overall incidence of TETs is 0.13 per 100,000 person-years in the US; however, it is higher among Asians (2). Previous studies have shown that patients with TETs have an elevated risk of developing a subsequent secondary tumor, indicating that certain genetic risk factors might be involved in the etiology of TET (2–4). The current knowledge about the biological properties of TETs is limited due to the low incidence. In particular, significant variability exists in the prognosis of TETs, indicating a complex heterogeneity among them. Previous studies investigated the etiology of TETs at the molecular level and mutations in EGFR, HER2, KIT, KRAS, and TP53 (5–13). However, discrepancies are found in the category and frequency of mutations in different studies.

The present study aimed to explore the genetic alterations and the possible therapeutic targets of TETs using next-generation sequencing (NGS) technology with 56 cancer-related hotspot genes. The correlation between gene mutations was analyzed using pathological classification, Masaoka–Koga stage classification, TNM stage, and overall survival (OS). In addition, the data on somatic mutations of TETs were retrieved from The Cancer Genome Atlas (TCGA) database and used to validate the findings. Finally, the literature was reviewed, and the genetic phenotypes of TETs were summarized. Thus, a better understanding of the molecular consequences of gene mutations might have therapeutic implications and support the personalized approach for the management of TETs.

## Materials and Methods

### Ethical Approval

The study was conducted following the ethical principles stated in the Declaration of Helsinki for medical research involving human participants. All participants provided written informed consent, and the ethical review board approved the study protocol for clinical research at the Tianjin Medical University General Hospital.

### Study Design

All patients who underwent surgical treatment or suffered from previous pathologically confirmed TETs at the Tianjin Medical University General Hospital between January 2011 and January 2019 were included in the study. Their clinicopathological characteristics are shown in **Table 1**. The pathological types and clinical staging were based on the 2015 WHO criteria and the Masaoka–Koga system (3, 4). Patients with TETs from the TCGA cohort (*n* = 123) were also employed in the present study to verify the findings. For the TCGA cohort, multidimensional data of gene expression and clinical information were obtained from cBioPortal (http://www.cbioportal.org/public-portal/). The gene mutation profile in both the cohorts was analyzed, and the prognostic values of TP53 and cyclin-dependent kinase inhibitor 2A (CDKN2A) were explored.

**Table 1:** Clinicopathological characteristics of study population from TCGA and our data.

		Our data			TCGA data		
		Type A, AB, B1, B2 (n=8)*	Type B3 (n=9)	Type C (n=17)	Type A, AB, B1, B2 (n=97)*	Type B3 (n=15)	Type C (n=11)
**Gender**	Male	6	8	13	53	6	4
	Female	2	1	4	44	9	7
**Age**	Median	58.5	54	55	57.5	62	65
	Range	33-73	39-60	16-66	17-84	40-71	44-78
**Smoking status**	Smoker	3	3	8	NP	NP	NP
	Non-smoker	5	6	9	NP	NP	NP
**Masaoka stage**	I	3	0	0	NP	NP	NP
	II	3	5	1	NP	NP	NP
	III	2	4	10	NP	NP	NP
	IV	0	0	6	NP	NP	NP
**TNM stage**	I	8	6	2	NP	NP	NP
	II	0	0	4	NP	NP	NP
	III	0	3	7	NP	NP	NP
	IV	0	0	4	NP	NP	NP
**Neoadjuavant therapy**	CT	0	0	0	2	0	0
	RT	0	1	0			
	CT+RT	0	0	0			
**Adjuavant therapy**	CT	0	1	6	26	8	5
	RT	1	6	1	1	0	2
	CT+RT	0	0	5	0	2	1

CT, hemotherapy; RT, radiotherapy; NP, Not provided.

*including mixed type, A/B1, B1/B2.

### Next-Generation Sequencing

DNA from the TETs was extracted using a QiAamp DNA FFPE tissue kit (Qiagen), and the DNA quality was evaluated according to the extent of DNA degradation. DNA extracted from the TET tissues was used for targeted capture sequencing of 56 cancer-associated genes (Lung core TM 56 genes; Burning Rock Biotech; **Supplementary Table 1**).

The concentration of the DNA samples was measured using the Qubit dsDNA assay to ensure that the genomic DNA was larger than 100 ng. The DNA was fragmented (average DNA fragment size of 180–220 bp), followed by hybridization with capture probe baits, hybrid selection with magnetic beads, and PCR amplification. A high-sensitivity DNA assay using a bioanalyzer was then used to assess the quality and size range. The available indexed samples were then sequenced using a NextSeq 500 bioanalyzer (Illumina, CA, USA) with paired-end reads. Flexbar software (version 2.7.0) was used for analyzing the raw data obtained from the NextSeq 500 runs to generate FASTQ data, trim the adapter sequences, and filter and remove the poor-quality reads (14). The sequencing depth was ~1000 units, and Varscan (v. 2.3) was used to call single-nucleotide variations and insertions/deletions with MAPQ >60, base quality >30, and allele frequency (AF) >1% (15).

True mutations were defined as variants that comprised >3 nonduplicated or >5 nonduplicated paired reads. The FASTQ data were mapped to the human genome (hg19) using BWAaligner 0.7.10 (http://bio-bwa.sourceforge.net/). Local alignment optimization, variant calling, and annotation were performed using GATK version 3.2 (https://www.broadinstitute.org/gatk/). DNA translocation analysis was performed using both Tophat2 (http://ccb.jhu.edu/software/tophat/index.shtml) and Factera version 1.4.3 (http://factera.stanford.edu). In the final step, to eliminate erroneous base calling and generate final mutation, variation frequency (>0.5%) was used and manual verification was performed using integrative Genomics Viewer version 2.3.72 (16–18).

### Mutation Prediction

PolyPhen-2 is an online prediction tool which could predict possible impact of amino acid changes of human proteins. We used PolyPhen-2 to predict the mutational consequence of missense mutations (16, 19). Three outcomes were used to show the prediction results: benign, possibly damaging, and probably damaging.

### Literature Review

Two individual researchers conducted platform searches on PubMed. Literature retrieval was performed through a combined search of the subject terms (“MeSH” on PubMed).

All available studies on patients with TETs who underwent NGS, which were published in English until May 01, 2021, were included, and the inclusion and exclusion criteria were listed. The inclusion criteria were as follows: (1) pathologically confirmed TETs, including thymomas and thymic carcinomas and (2) NGS performed for thymic epithelial tumors. The exclusion criteria were as follows: (1) studies with a design of literature review, systematic review, basic research, letter to editors, diagnostic study, and so on, (2) studies using the PCR sequencing method, and (3) studies using repeated patient cohorts with another study. No limitations were imposed on the nationalities of the participants.

### Statistical Analysis

The gene mutation status was compared with the patient’s clinicopathological characteristics using the Fisher’s exact test and the Wilcoxon–Mann–Whitney test. Survival analysis was calculated using the Kaplan–Meier method to perform the log-rank test and two stage hazard rate comparison when the curves crossed using softwares GraphPad Prism 7.0 (GraphPad Software, CA, USA) and R version 3.6.1 (cran.r-project.org) (20). A two-sided statistically significant cutoff was set at *P <*0.05.

## Results

### Population Study

A total of 17 thymoma (type A, *n* = 3; type AB, *n* = 2; typeB1, *n* = 2; type B1/B2, *n* = 1; type B3, *n* = 9) and 17TCs were collected in this study. The distributions of sex and age were similar between the two groups. The patients with TCs+type B3 TETs presented with an advanced Masaoka–Koga stage compared with the other types (**Table 1**). For patients with TETs from the TCGA cohort, 123 patients underwent whole-genome sequencing, including 97 patients with types A, AB, B1, and B2, 15 patients with type B3, and 11 patients with TCs. However, some information such as smoking status and Masaoka–Koga stage was not provided (**Table 1**).

### Genetic Mutations in TETs

**Figure 1:**
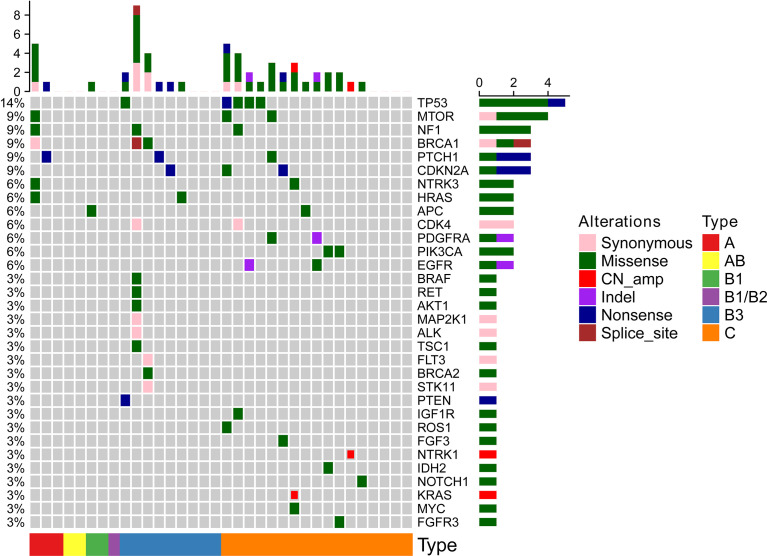
The mutational results of all 34 TETs in our cohort.

**Figure 2:**
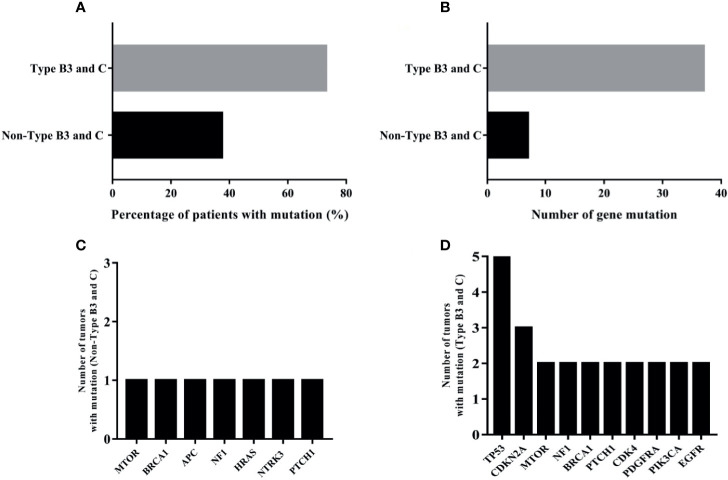
The analysis of mutational results of TETs in our cohort; **(A)**: the mutation percentage in type B3 and C and non-type B3 and C TET patients; **(B)**: the number of mutated genes in type B3 and C and non-type B3 and C TET patients; **(C)**: the numbers of tumors with mutation of seven genes in type A and B1/B2 thymomas; **(D)**: ten most frequently mutational genes in type B3/C TETs patients.

All 34 TETs underwent genetic mutation analysis with a panel of 56 cancer-related genes. Among the 34 TETs, 22 tumors were detected with at least one gene mutation (non-TCs+type B3 TETs, *n* = 3; type B3, *n* = 6; TCs, *n* = 13), and the most frequent gene mutations were TP53 (*n* = 5), MTOR (*n* = 3), BRCA1 (*n* = 3), NF1 (*n* = 3), CDKN2A (*n* = 3), and PTCH1 (*n* = 3) (**Figure 1**). Seven out of 26 patients with TCs+type B3 TETs and 5 out of 8 patients with type A/B1/B2 thymoma had no detected gene mutations. The mutation percentages were 73.08% for patients with types TCs+type B3 TETs and 37.50% for patients with types A/B1/B2 (**Figure 2A**). In addition, the number of mutated genes was significantly higher in patients with type TCs+type B3 TETs than in patients with type A/B1/B2 thymoma (typeTCs+type B3 TETs = 33 *vs* type A + B1/B2 = 7) (**Figure 2B**). For patients with types A and B1/B2 thymoma (*n* = 9), seven gene mutations, including MTOR, BRCA1, APC, NF1, HRAS, NTRK3, and PTCH1, were detected, and each gene appeared only once in patients with non-TCs+type B3 TETs (**Figure 2C**). For patients with type B3/C (*n* = 26), 33 gene mutations were found and the most frequent mutations were TP53 (*n* = 5), followed by CDKN2A (*n* = 3), MTOR (*n* = 2), NF1 (*n* = 2), BRCA1 (*n* = 2), PTCH1 (*n* = 2), CDK4 (*n* = 2), PDGFRA (*n* = 2), PIK3CA (*n* = 2), and EGFR (*n* = 2) (**Figure 2D**). Importantly, all TP53 or CDKN2A mutations were seen in type TCs+type B3 TETs only (**Figure 1**). There are 18 patients with missense mutations. The prediction results of PolyPhen-2 were showed in the **Supplementary Figure 2** which indicated that all of TP53 missense mutations in our cohort were probably damaging.

The mutations in the 6 most frequently mutated genes in the cohort were further validated using a cohort of 123 patients with TETs from the TCGA database. The mutation types are shown in **Supplementary Figure 1**. TP53 was also the most frequent mutation in the TCGA cohort similar to that in the cohort. CDKN2A (*n* = 6) was a highly frequent mutation, followed by NF1 (*n* = 3), MTOR (*n* = 1), BRCA1 (*n* = 1), and PTCH1 (*n* = 1). The mutation characteristics of the six genes are listed in **Table 2**.

**Table 2:** Thymic epithelial tumor patients with high frequent gene alterations (somatic mutation and copy number alterations) in our cohort and TCGA data.

		Percentage (No.)	Type	Mutation classification
**TP53**	Our data	14.3% (5)	Type B3, n=1	Missense variant, n=4
			Type C, n=4	Nonsense variant, n=1
	TCGA data	5% (6)	Type A, n=1	Missense variant, n=3
			Type AB, n=1	Deletion variant, n=1
			Type B2, n=1	CN-del, n=2
			Type B3, n=1	
			Type C, n=2	
**MTOR**	Our data	8.6% (3)	Type A, n=1	Missense variant, n=3
			Type C, n=2	
	TCGA data	0.8% (1)	Type C, n=1	Missense variant, n=1
**BRCA1**	Our data	8.6% (3)	Type A, n=1	Missense variant, n=1
			Type B3, n=2	Splice-site, n=1
				Synonymous variant,n=1
	TCGA data	0.8% (1)	Type C, n=1	CN-amp, n=1
**NF1**	Our data	8.6% (3)	Type A, n=1	Missense variant, n=3
			Type C, n=1	
	TCGA data	2.4% (3)	Type A, n=2	Missense variant, n=2
			Type C, n=1	Nonsense variant, n=1
**CDKN2A**	Our data	8.6% (3)	Type B3, n=1	Missense variant, n=1
			Type C, n=2	Nonsense variant, n=2
	TCGA data	5% (6)	Type A, n=1	Deletion variant, n=1
			Type AB, n=1	CN-del, n=5
			Type B3, n=2	
			Type C, n=2	
**PTCH1**	Our data	8.6% (3)	Type A, n=1	Missense variant, n=1
			Type B3, n=1	Nonsense variant, n=2
			Type C, n=1	
	TCGA data	0.8% (1)	Type AB, n=1	Missense variant, n=1

CN-amp, Copy number variation-amplification.

CN-del, Copy number variation-deletion.

Total patient number: Our data, n=35; TCGA data, n=123.

Among the 123 patients with TETs from the TCGA cohort, the most frequent gene mutations were GTF2I (*n* = 49), HRAS (*n* = 10), TTN (*n* = 8), MUC16 (*n* = 6), UNC93B1 (*n* = 5), MUC4 (*n* = 5), NPIPA2 (*n* = 4), TP53 (*n* = 4), ZNF208 (*n* = 3), and BCOR (*n* = 3) (**Supplementary Table 2**). Also, the top 10 highly frequent somatic gene mutations in patients with non-TCs+type B3 TETs and type TCs+type B3 TETs were also listed and compared (**Figure 2** and **Supplementary Tables 3** and **4**). In the TCGA cohort, TP53 had the highest gene mutation in patients with TCs+type B3 TETs compared with non-TCs+type B3 TETs, which was concordant with that in the cohort.

Furthermore, the basic characteristics of TP53 somatic mutations in patients from the present cohort and the TCGA cohort were summarized. Most TP53 somatic mutations were missense mutations, while nonsense and deletion mutations were detected once in the present cohort and TCGA cohort, respectively (**Table 3**).

**Table 3:** Frequency of different TP53 somatic mutations in Thymoma patients from our and TCGA cohort.

Our cohort			TCGA		
AA change	Type	#Mut	AA change	Type	#Mut
G244D	Missense	1	D281Afs*64	Deletion	1
E349*	Nonsense	1	R273C	Missense	1
R282P	Missense	1	L194R	Missense	1
F113C	Missense	1	R248L	Missense	1
R248L	Missense	1			

*stop codon.

#Frequency of mutations.

### Survival Analysis

**Figure 3:**
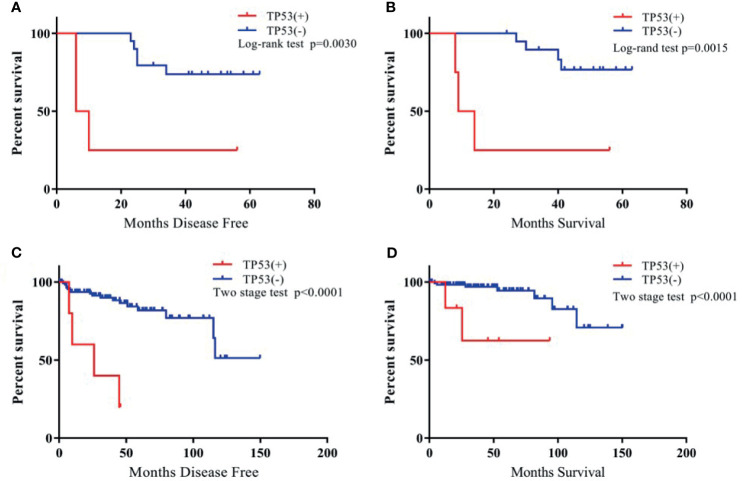
The Kaplan-Meier survival curve of TP53(+) *vs.* TP53(-) TET patients. **(A)**: TP53(+) *vs*. TP53 (-) TET patients of DFS in our cohort; **(B)**: TP53(+) *vs.* TP53 (-) TET patients of OS in our cohort; **(C)**: TP53 (+) *vs.* TP53 (-) TET patients of DFS in TCGA cohort; **(D)**: TP53 (+) *vs*. TP53 (-) TET patients of OS in TCGA cohort.

The gene with the highest frequency of mutations among patients with TETs from the TCGA cohort, including TP53, CDKN2A, and NF1, were selected, and their roles in the prognosis of patients with TETs were investigated. In the cohort of patients with thymoma from the hospital, the most frequent mutation was TP53. All patients with TP53 mutations were classified as Masaoka–Koga stage III or IV and received postoperative radiotherapy or chemotherapy. Using log-rank tests or two stage hazard rate comparison, the study found that the patients with TP53 mutations in the cohort of the hospital showed a significantly shorter disease-free survival (DFS) and overall survival (OS) compared with those without TP53 mutation (**Figure 3**). In addition, patients with CDKN2A (a tumor suppressor gene) mutations in the present cohort exhibited a trend of poor survival compared with those without CDKN2A mutations. However, the difference was not significant, probably due to limited patient numbers (**Supplementary Figure 3A**). The survival analysis between NF1(+) and NF1(–) TETs was also performed, and the results indicated that the NF1(–) TETs had a better survival rate (**Supplementary Figure 4**).

In addition, this study also investigated TP53, CDKN2A, and NF1 mutations and explored the relationship between individual gene mutations and DFS and OS in patients in the TCGA cohort. Further, 50% of TP53 mutations and 66.7% of CDKN2A mutations were of TCs+type B3 TETs (**Table 2**). The study confirmed, using the TCGA dataset, significantly shorter DFS and OS for TETs with TP53 mutations (**Figure 3**) and a trend of shorter DFS and OS for TETs with CDKN2A mutations (**Supplementary Figure 3B**). NF1 mutation indicated significantly poor survival in patients with TETs from the present cohort; however, NF1 mutation had no correlation with the prognosis of patients with thymoma in the TCGA cohort (**Supplementary Figure 4**). Moreover, the study also investigated the relationship between nine other most frequent gene mutations from the TCGA dataset and the prognosis of thymoma. However, none of the other gene mutations in the TCGA cohort exhibited a significant correlation with the prognosis of patients with thymoma (**Supplementary Figure 5**).

## Discussion

The underlying molecular and genetic mechanisms of TETs are yet to be fully elucidated due to their low incidence and histological heterogeneity compared with other thoracic malignancies (8–12). The findings of previous studies on the molecular characteristics of TETs have been inconsistent, and very few studies focused on the genetic alterations in Asian patients (6–10, 12, 17, 21).

The present study, based on an NGS 56–cancer gene panel, found that TETs with types AB1 and B2 exhibited a remarkable difference in somatic gene mutations compared with types B3 and C, in terms of mutation percentage and frequency. TP53 was the most frequent gene mutation in all 34 patients with TETs from the present cohort, and more importantly, TP53 and CDKN2A mutations were detected only in patients with types B3 and C. Although the sequencing methods and profiling in the TCGA cohort and the present cohort were not exactly the same, TP53 and CDKN2A mutations were found to be more common in patients with TCs+type B3 TETs (TP53, 50%; CDKN2A, 66.7%, in TCs+type B3 TETs) in the TCGA cohort. Survival analysis from both the TCGA cohort and the present cohort demonstrated that TP53 mutations indicated a significantly worse prognosis in patients with TETs, and previous studies also proved this (22–24). The patients with CDKN2A mutations also exhibited a trend of poor survival compared with those without CDKN2A mutations; however, this difference was not significant. Previous studies reported the mutation frequency of CDKN2A in thymic carcinomas were 11%-35% and most of them were truncating mutation (22, 23, 25). Further studies with larger sample sizes are necessary to validate the role of CDKN2A mutations in the prognosis of TETs.

A comprehensive literature review was performed, and the genetic sequencing data were summarized to further explore the molecular and biological mechanisms of TETs. The clinical characteristics and high-frequency gene mutations are listed in **Table 4**, comprising 15 studies that included 797 TETs (465 thymomas and 332 thymic carcinomas) (25–39). All 15 studies were published between 2009 and 2020, and DNA-based NGS with different gene panel sizes was used. As shown in **Table 4**, as the number of genes for sequencing increased, more gene mutations were detected. In 6 out of 15 studies, TP53 was the most frequent mutation in thymic carcinomas, and the mutation frequency ranged from 7.7% to 26.7%. However, the mutation of TP53 in thymomas was rare. This was consistent with the findings of the present study that TP53 was the gene mutation with the highest mutation frequency (23.5%) in TCs.

**Table 4:** Gene mutation analysis of TETs from previously published literature.

Case	Author	Year	Type	N0	Mutation	Sequencing Method	Country
1	Chen et al. (26)	2020	Thymoma	50	MAP3K1 (98%), TGFBR2 (96%), KMT2C (94%), ARID1A (92%), PRKDC (90%)	Next-generation sequencing for 315 genes	China
			Thymic carcinoma	5	ARID1A (100%), KMT2C (100%), MAP3K1 (100%)		
2	Thompson et al. (27)	2020	Thymoma	3	HRAS (33.3%)	Next-generation sequencing for 1385 genes	USA
3	Sakane et al. (28)	2019	Thymoma	33	HRAS (3.0%); PIK3CA (6.1%); AKT1 (3.0%)	Single-base extension multiplex assay	Japan
			Thymic carcinoma	54	KRAS (11.1%); HRAS (5.6%); TP53 (9.3%); EGFR (3.7%); PIK3CA (1.9%); NRAS (1.9%); AKT1 (1.9%)		
4	Enkner et al. (29)	2017	Type A thymoma	18	HRAS (16.7%)	Next-generation sequencing for 50 genes	Austria
			Type B3 thymoma	19	SMARCB (5.3%); STK11 (5.3%)		
			Thymic carcinoma	35	TP53 (25.7%); CDKN2A (11.4%); FGFR3 (5.7%); KIT (5.7%); ALK (2.9%); ATM (2.9%); ERBB4 (2.9%); NRAS (2.9%);		
5	Saito et al. (30)	2017	Thymic carcinoma	10	TET2 (30%); CACNA1A (30%); HTT (20%); MYNN (20%); OR5T2 (20%); ARID1B (20%); CYLD (20%); SETD2 (20%);	Whole exome sequencing	Japan
6	Asao et al. (31)	2016	Thymic carcinoma	52	TP53 (7.7%), KRAS (3.8%), FBXW7 (3.8%), NRAS (1.9%),	Next-generation sequencing for 50 genes	Japan
7	Song et al. (32)	2016	Thymoma	37	EGFR (2.7%), PIK3CA (2.7%);	Next-generation sequencing for 22 genes	China
			Thymic carcinoma	15	PIK3CA (6.7%)		
8	Moreira et al. (25)	2015	Type B3 thymoma	6	BCOR (50%); MLL3 (16.7%)	Next generation sequencing	USA
			Thymic carcinoma	15	TP53 (26.7%), SMAD4 (13.3%), and CYLD (13.3%), KDM6A (20%), SETD2 (13.3%), MLL3 (13.3%), MLL2 (13.3%).		
9	Petrini et al. (33)	2014	Thymoma	38	GTF2I (42.1%); TP53 (5.3%); ALK (5.3%); PPP2RIA (5.3%)	Exome sequencing or 197-gene assay	USA
			Thymic carcinoma	16	TP53 (25%); CYLD (18.8%); BAP (12.5%); PBRM (12.5%); CDKN2A (12.5%)		
10	Shitara et al. (34)	2014	Thymic carcinoma	12	NF1 (16.7%); 8.3% for HRAS, PBRM1, DDR2, ASXL1, CDK8, CDKN2A, DCC, IGF1R, IKBKE, KAT6B, KDM6A, KIT, KMT2A, KMT2D, NKX2-1, PAX5, PDGFRA, PKHD1, ROS1, RUNX1T1, SMARCA4, TET1, TP53;	Ion Torrent next-generation sequencing for 409 cancer-related genes	Japan
11	Wang et al. (30)	2014	Thymoma	31	3.2% for ASXL1, DCC, EGFR, ERG, HRAS, MAGI1, PDGFRA, PRCC, PTGS2, RUNX1, SDHA, SETD2, SRC, TET2, TP53	Massively parallel sequencing of 197 cancer-related genes.	USA
			Thymic carcinoma	47	TP53 (25.5%); BAP1 (10.6%); CYLD (8.5%); KIT (8.5%); DNMT3A (8.5%); SETD2 (8.5%); TET3 (6.4%); 4.3% for ASXL1, BRCA2, CDKN2A, DCC, SMARCA4 and WT1.		
12	Girard et al. (35)	2009	Thymoma	38	KRAS (2.6%); HRAS (2.6%)	Array-based comparative genomic hybridization.	USA
			Thymic carcinoma	7	KIT (28.6%); KRAS (14.3%)		
13	Asselta et al. (36)	2021	Thymic carcinoma	15	FGFR3(33.3%);CDKN2A(20%);SMARCB1(13.3%); 6.6% for ATM, NRAS, SRC, APC, KIT, MET	Next-generation sequencing for 50 genes	Italy
14	Massoth et al. (37)	2020	Thymoma	242	KMT2A-MAML2 Fusion (4%)	Next-generation sequencing	USA
15	Sakane et al. (38)	2021	Thymic carcinoma	54	TP53 (18.5%), KIT (7.4%), and PDGFRA (5.6%)	Next-generation sequencing for 50 genes	Japan

The malignant potential of type B3 TETs, especially in an advanced stage, shows a poor prognosis, even similar to that of TCs. Hence, TCs+type B3 TETs were classified together in the present study. The sequencing analysis indicated that the gene mutations and frequency differed between TCs+type B3 TETs and non-TCs+type B3 TETs. Previous studies also focused on the difference between thymomas and TCs. However, most of these studies classified type B3 and types A/B1/B2 together, not with TCs Only a study by Enkner et al. separated type B3 from other thymomas (types A/B1/B2) and reported that the mutations between type TCs+type B3 TETs and non-TCs+type B3 TETs were very different (29). Other studies that compared the molecular mechanisms between type B3 TETs and TCs found comparable gene mutations with similar frequencies. The present genetic analysis found that types B3 and TCs exhibited similar gene mutations, including TP53. Hence, placing type B3 and TCs together was suggested to be more appropriate. Previous studies reported that TP53 mutations in TETs were associated with more aggressive behavior (5, 12, 13, 17, 40).

In the present cohort and the TCGA cohort, patients with TETs having TP53 mutations had significantly poorer survival compared with those without TP53 mutations. HRAS mutations, which were detected in TETs in the present study, were detected in previous studies as well. According to the literature review, five studies reported that the mutations of HRAS in TETs and their frequencies were very inconsistent, ranging from the lowest of 2.6% to the highest of 33.3% (27–30, 34, 35). Furthermore, four studies reported that the frequency of CDKN2A mutations ranged from 4.3% to 12.5%. This study confirmed that CDKN2A was a common mutation in the present cohort, with a frequency of 11.8% in thymic carcinomas, which was similar to that in previous studies. The study also found that TETs with CDKN2A mutations exhibited a trend of poor survival compared with those without CDKN2A mutations; however, this was not statistically significant, probably due to the small sample size.

The effect of CDKN2A on the prognosis of TETs needs further investigation. Another gene with a relatively frequent mutation in TETs was NF1, with mutation frequencies of 8.6% and 5% in the present cohort and the TCGA cohort, respectively. However, Shitara reported that 16.7% of the TETs exhibited NF1 mutations in their cohort study (34). The difference in sample size and histological distribution might have resulted in this discrepancy.

In TCGA cohort we found that GTF2I is the gene mutation with the highest mutation frequency in TETs. Previous studies also reported that GTF2I is the most frequently mutated gene in thymomas especially in type A and type AB TETs, however its frequency is lower than other types thymomas and thymic carcinomas (41–43). It was reported that thymomas had a unique GTF2I mutation Leu404His which was not found in other tumors (42). TETs with GTF2I mutation had better prognosis and our analysis also demonstrated the similar trend (41).

Moreover, this study had some limitations. First, the gene panel of NGS was relatively too small to thoroughly explore the genetic mechanism of TETs. In addition, previous studies also reported some gene mutations with a high frequency, which were not seen in the present cohort, such as GTF2I, CYLD, SMAD4, and a few others. However, the function and value of these genes in the prognosis of TETs are unknown and need to be further investigated. Finally, the sample sizes in the present cohort and the TCGA cohort were small, especially given the heterogeneous histology of TETs.

## Conclusion

Our study found that the gene mutations between TCs+type B3 TETs and non-TCs+type B3 TETs were drastically different. The mutations in TP53 were more frequent in type B3/C TETs, indicating a worse prognosis. Targeted therapy against TP53 might be an effective strategy for treating thymic carcinomas. However, further validation is needed through prospective clinical studies with a larger sample size.

## Data Availability Statement

The original contributions presented in the study are included in the article/**Supplementary Material**. Further inquiries can be directed to the corresponding authors.

## Ethics Statement

The studies involving human participants were reviewed and approved by Tianjin Medical University General Hospital. The patients/participants provided their written informed consent to participate in this study.

## Author Contributions

SX, XFL, and HZ retrieved and analyzed all of the data in the study. SX, XFL, HZ, LZ, SZ, XL, LY, TS, and ZS revised the manuscript for important intellectual contents. SX and JC designed, checked, and supervise all study process. All authors contributed to the article and approved the submitted version.

## Funding

The present study was funded by the National Natural Science Foundation of China (No. 81772464) and Tianjin Science and Technology Plan Project (19ZXDBSY00060).

## Conflict of Interest

The authors declare that the research was conducted in the absence of any commercial or financial relationships that could be construed as a potential conflict of interest.

## Supplementary Material

Supplementary Figure 1 Six genes alterations in TCGA.

Supplementary Figure 2 The heatmap of mutations prediction via PolyPhen-2.

Supplementary Figure 3 The Kaplan-Meier survival curve of CDKN2A(+) vs. CDKN2A(-) TET patients. A: CDKN2A(+) vs. CDKN2A(-) TET patients of DFS in our cohort; B: CDKN2A(+) vs. CDKN2A(-) TET patients of OS in our cohort; C: CDKN2A(+) vs. CDKN2A(-) TET patients of DFS in TCGA cohort; B: CDKN2A(+) vs. CDKN2A(-) TET patients of OS in TCGA cohort.

Supplementary Figure 4 The Kaplan-Meier survival curve of NF1(+) vs. NF1 (-) TET patients. A: NF1 (+) vs. NF1 (-) TET patients of DFS in our cohort; B: NF1 (+) vs. NF1 (-) TET patients of OS in our cohort; C: NF1 (+) vs. NF1 (-) TET patients of DFS in TCGA cohort; B: NF1 (+) vs. NF1 (-) TET patients of OS in TCGA cohort.

Supplementary Figure 5 The Kaplan-Meier survival curve of GTF2I(+) vs. GTF2I(-), HRAS(+) vs. HRAS(-), MUC4(+) vs. MUC4(-), MUC16(+) vs. MUC16(-), TTN(+) vs. TTN(-), UNC93B1(+) vs. UNC93B1(-), BCOR(+) vs. BCOR(-), NPIPA2(+) vs. NPIPA2(-), ZNF208(+) vs. ZNF208(-) TET patients in TCGA cohort.

Supplementary Table 1 The lists of 56 cancer-associated genes.

Supplementary Table 2 The gene somatic mutation distribution of TCGA all types of TETs patients.

Supplementary Table 3 The gene somatic mutation distribution of TCGA type B3 and C patients.

Supplementary Table 4 The gene somatic mutation distribution of TCGA non-B3 and C patients.
