# Supplementary figures and images for: Frequent Genetic Alterations and Their Clinical Significance in Patients With Thymic Epithelial Tumors
Source: Front Oncol. 2021 Jul 8;11:667148. doi: 10.3389/fonc.2021.667148 (PMC8296820; doi:10.3389/fonc.2021.667148)

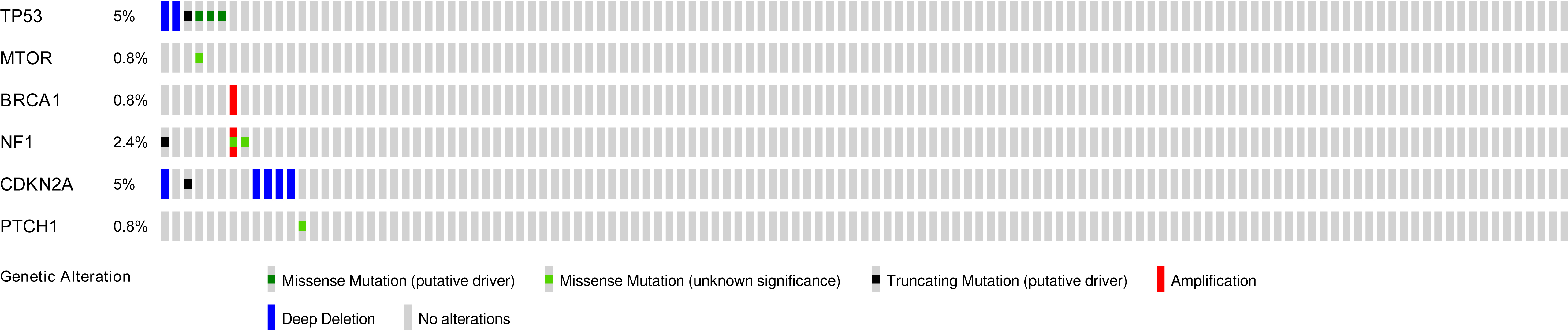

Supplement: Supplementary Figure 1 — Six genes alterations in TCGA. [file Image_1.jpg]

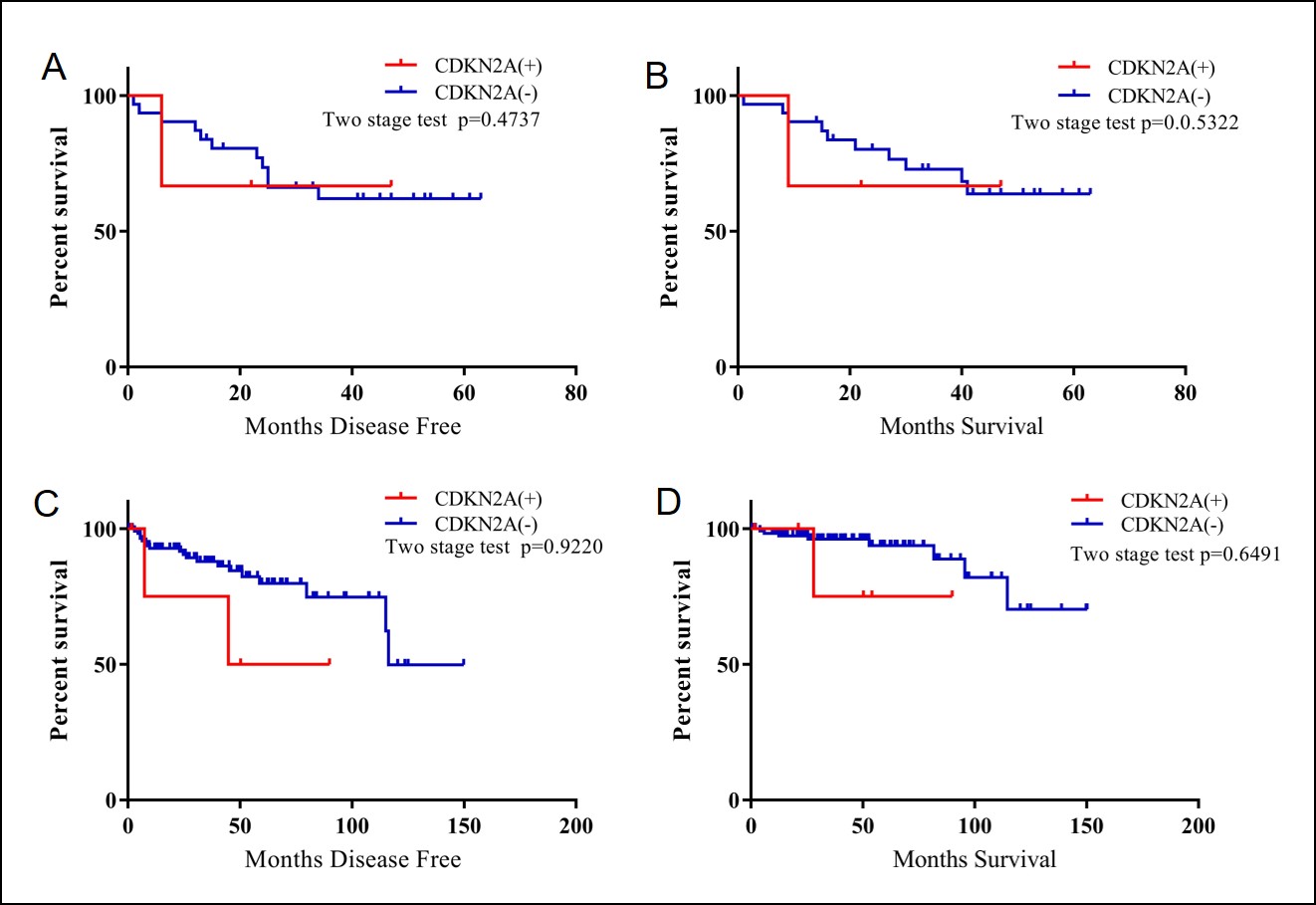

Supplement: Supplementary Figure 2 — The heatmap of mutations prediction via PolyPhen-2. [file Image_2.jpg]

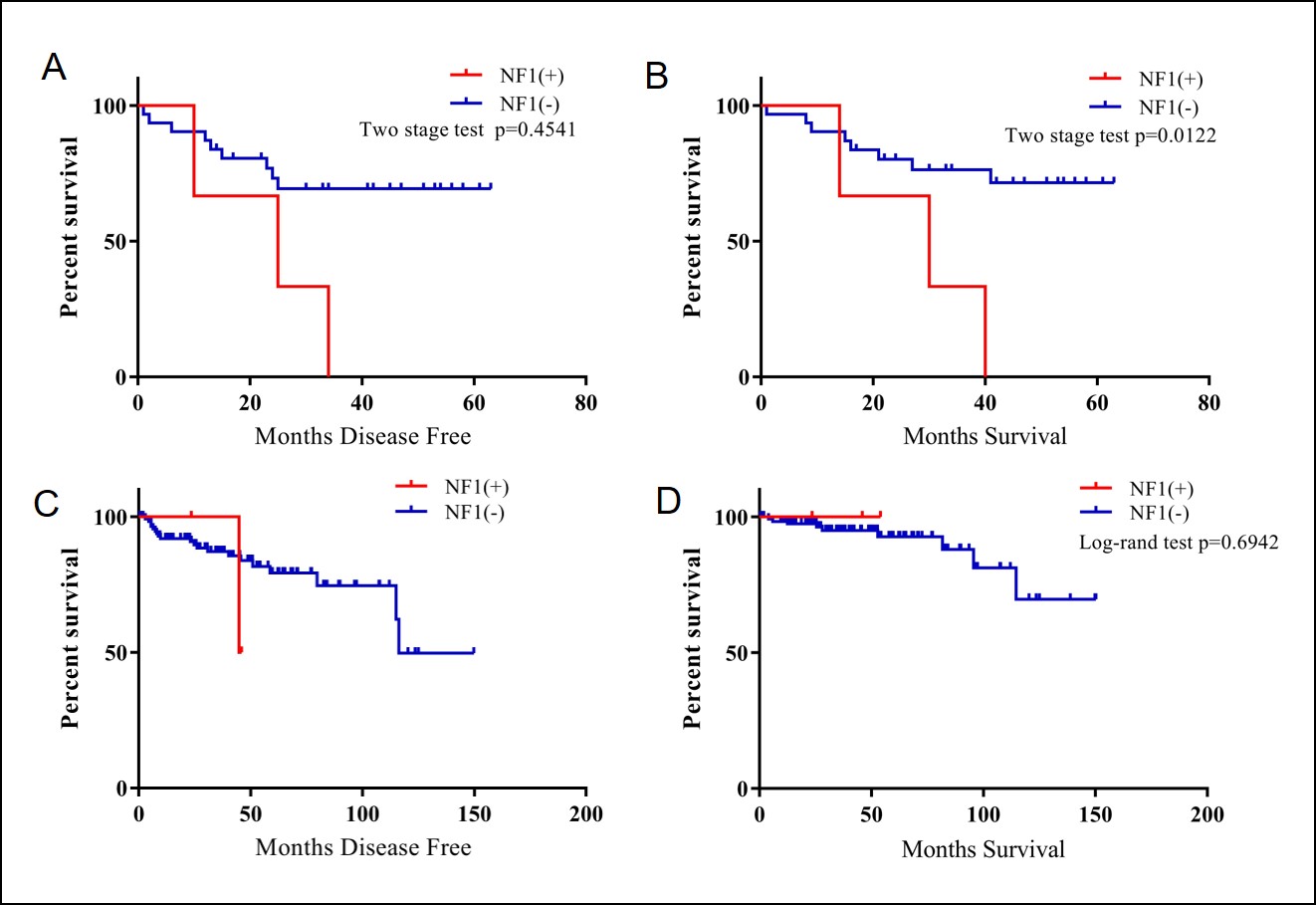

Supplement: Supplementary Figure 3 — The Kaplan-Meier survival curve of CDKN2A(+) vs. CDKN2A(-) TET patients. A: CDKN2A(+) vs. CDKN2A(-) TET patients of DFS in our cohort; B: CDKN2A(+) vs. CDKN2A(-) TET patients of OS in our cohort; C: CDKN2A(+) vs. CDKN2A(-) TET patients of DFS in TCGA cohort; B: CDKN2A(+) vs. CDKN2A(-) TET patients of OS in TCGA cohort. [file Image_3.jpg]

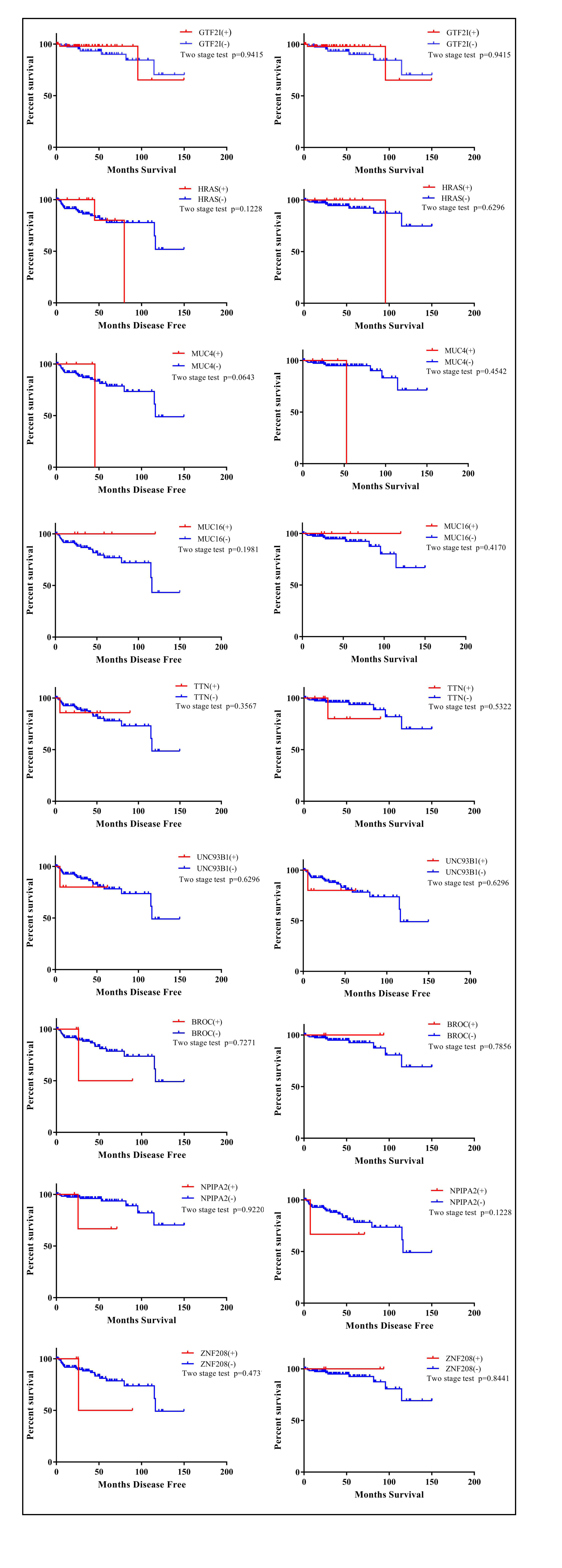

Supplement: Supplementary Figure 4 — The Kaplan-Meier survival curve of NF1(+) vs. NF1 (-) TET patients. A: NF1 (+) vs. NF1 (-) TET patients of DFS in our cohort; B: NF1 (+) vs. NF1 (-) TET patients of OS in our cohort; C: NF1 (+) vs. NF1 (-) TET patients of DFS in TCGA cohort; B: NF1 (+) vs. NF1 (-) TET patients of OS in TCGA cohort. [file Image_4.jpg]
