# Supplementary material for: Frequent Genetic Alterations and Their Clinical Significance in Patients With Thymic Epithelial Tumors
Source: Front Oncol. 2021 Jul 8;11:667148. doi: 10.3389/fonc.2021.667148 (PMC8296820; doi:10.3389/fonc.2021.667148)
Supplement: Supplementary Table 1 — The lists of 56 cancer-associated genes. [file Table_1.docx]

**Supplementary table 1. Gene mutation profile**

| AKT1 | ALK | ARAF | ATM | BIM | BRAF | BRCA1 | BRCA2 |
| --- | --- | --- | --- | --- | --- | --- | --- |
| CCND1 | CDK4 | CDK6 | CDKN2A | CYP2D6 | CTNNB1 | DDR2 | DPYD |
| EGFR | ERBB2 | ERBB3 | ERBB4 | FGF19 | FGF3 | FGF4 | FGFR1 |
| FGFR2 | FGFR3 | FLT3 | HRAS | JAK1 | JAK2 | KDR | KIT |
| KRAS | MYC | MAP2K1 | MET | MTOR | NRAS | NRG1 | NTRK1 |
| NTRK2 | NTRK3 | PDGFRA | PIK3CA | PTCH1 | PTEN | RB1 | RAF1 |
| RET | ROS1 | SMO | STK11 | TP53 | TSC1 | TSC2 | UGT1A1 |

**Mutation &CNV Merge&mutation&CNV SNP**
